# Current insights on m6A RNA modification in acute leukemia: therapeutic targets and future prospects

**DOI:** 10.3389/fonc.2024.1445794

**Published:** 2024-11-12

**Authors:** Parminder Kaur, Pankaj Sharma, Prateek Bhatia, Minu Singh

**Affiliations:** Haematology-Oncology Unit, Department of Paediatrics, Postgraduate Institute of Medical Education and Research, Chandigarh, India

**Keywords:** RNA modification “writers”, RNA modification “readers”, RNA modification “erasers”, acute leukemia, N6-methyladenosine (m6A)

## Abstract

RNA modification is the critical mechanism for regulating post-transcriptional processes. There are more than 150 RNA modifications reported so far, among which N6-Methyladenosine is the most prevalent one. M6A RNA modification complex consists of ‘writers’, ‘readers’ and ‘erasers’ which together in a group catalyze, recognize and regulate the methylation process of RNA and thereby regulate the stability and translation of mRNA. The discovery of erasers also known as demethylases, revolutionized the research on RNA modifications as it revealed that this modification is reversible. Since then, various studies have focused on discovering the role of m6A modification in various diseases especially cancers. Aberrant expression of these ‘readers’, ‘writers’, and ‘erasers’ is found to be altered in various cancers resulting in disturbance of cellular homeostasis. Acute leukemias are the most common cancer found in pediatric patients and account for 20% of adult cases. Dysregulation of the RNA modifying complex have been reported in development and progression of hematopoietic malignancies. Further, targeting m6A modification is the new approach for cancer immunotherapy and is being explored extensively. This review provides detailed information about current information on the role of m6A RNA modification in acute leukemia and their therapeutic potential.

## Graphical Abstract

M6A RNA modifications: emerging drivers of leukemia growth and progression.

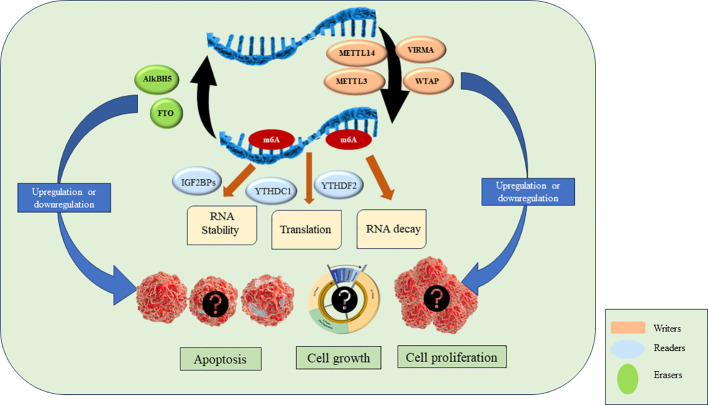

## Introduction

Recent studies have identified RNA modifications as one of the key post-transcriptional regulatory mechanisms in cancer. There are more than 150 types of RNA modifications reported till date. The most abundant RNA modification is the N6-Methyladenosine (m6A) where methylation takes place at the 6^th^ N of adenylate of RNA on the consensus sequence DRACH (D = G, A, or U; R = G or A; H = A, C, or U) (1). This modification is found in the non-coding RNA, messenger RNA, transfer RNA, small nucleolar RNA, microRNA, and ribosomal RNA. It is most abundantly found at the exons, stop codons, and 3’ UTR regions of mRNA (2, 3). M6A modification is catalyzed by methyltransferase complex and is erased to maintain cellular homeostasis. A set of proteins recognized as ‘writers’, ‘erasers’, and ‘readers’ are involved in methylation, demethylation, and signaling pathways respectively (4). Writers consist of methyltransferase complex’s which contains Methyltransferase-like 3 (METTL3), Methyltransferase-like 14 (METTL14), Wilms tumor-1 associated protein (WTAP), Vir-like m6A methyltransferase associated (VIRMA), Zinc finger CCCH-type containing 13 (ZC3H13), RNA-binding protein 15/15B (RBM15/15B), HAKAI (CBLL1) (5). They help to achieve the methylation of RNA molecule. Readers recognize the methylation to regulate the translation and stability of RNA and play an important role in various signaling pathways. YTH domain-containing family protein 1/2/3 (YTHDF1/2/3), YTH domain-containing protein 1/2 (YTHDC1/2), Insulin-like growth factor-binding protein 1/2/3 (IGF2BP1/2/3), Heteronuclear ribonucleoproteins (including HNRNPC, HNRNPG and HNRNPA2B1) are various readers reported so far. Protein categorized under the Erasers category reverses the methylation modification i.e., carry out the demethylation reaction. Fat mass and obesity-associated protein (FTO/ALKBH9) and AlkB homolog 5 (ALKBH5) are two demethylase proteins identified so far (6). These writers, readers and erasers work in a coordinated manner to achieve homeostasis in the body. Any alteration in the function of these may result in the disruption of biological processes causing various diseases including cancers.

There are multiple reports on the development of cancer due to dysregulation of RNA modifications and their aberrant expression is linked to the initiation, progression, and drug resistance in various cancers. Acute leukemia is the most common pediatric malignancy and accounts for 20% adult cancers. Acute lymphoblastic leukemia (ALL) and acute myeloid leukemia (AML) are the two major types of acute leukemia depending on the lineage of cells involved and ALL is further categorized as T-cell and B-cell ALL. With the advancement of various molecular and genetic techniques, the outcome of ALL is significantly improved over the years, leading to better survival rates and more effective treatment regimens. However, despite these advancements, there is still a pressing need for new therapies to tackle relapse, chemoresistance, and to improve outcomes for high-risk patients who do not respond well to standard chemotherapy protocols. The challenge lies in the heterogeneity of the disease, with different genetic mutations and molecular pathways contributing to its aggressiveness and resistance to treatment.

Further, targeting m6A modification regulators is a new approach to cancer immunotherapy and is being widely explored in various cancers. Targeting FTO with inhibitors such as Meclofenamic acid has shown to have inhibitory effects on cell proliferation and metastasis in breast cancer and glioblastoma cells (7). Similar results are also seen on targeting ALKBH5 in AML (8). Moreover, targeting methyltransferase complex with STM2457 has been shown to inhibit the growth of AML cells (9). Thus, targeting the RNA modification regulatory pathways may pave the way for the development of novel therapeutic strategies for the treatment of hematopoietic malignancies. This review summarizes the recent information on the role of m6A RNA modification in pathogenesis of acute leukemia and its therapeutic potential.

## Key players of m6A modification: discovery and functions

The first report of methylation on mRNA was published in 1974 by Desrosiers et al. in Novikoff hematoma cells (10). However, the writer complex was first purified after two decades in 1994 from HeLa cell extracts which provided insights into the mechanisms of formation of m6A modification. The latter study showed that methyltransferase contains three subunits MT-A1 (30 kDa), MT-A2 (200 kDa) and MT-B (875 kDa) (11). Further studies by the same research group showed that MT-A contains the AdoMet binding site on a 70-kDa subunit. Following that, expression studies in bacteria and antisera production from rabbits revealed that MT-A70 is a critical subunit of methyltransferase complex and Northern blotting studies showed that MT-A70 mRNA undergoes alternative splicing and is present abundantly in human tissues (12). Further components of methyltransferase complex were not known earlier due to the lack of advanced approaches. It was in 2014 when Schwartz et al. using a proteomic approach identified WTAP, KIAA1429 and METTL14 as important components of methyltransferase complex (13). Simultaneously, WTAP and METTL14 were published by two other groups while KIAA1429 could not be identified in any subsequent studies (14, 15). WTAP was shown to act as a regulatory subunit that interacts with METTL3-METTL14 heterodimer and was required for the localization of complex to nuclear speckles (15). The crystal structure of the METTL3-METTL14 complex was explained in 2016 by Wang et al. which revealed METTL3 is the catalytic component (16). The other writer VIRMA was reported as a part of the methyltransferase complex in 2018 which helps in mediating mRNA methylation at 3′UTR and near the stop codon (17). The writer ZC3H13 was reported by Wen et al., as a critical regulator of methyltransferase complex (18). Another recently identified writer HAKAI was shown to stabilize the core components of the methyltransferase complex (19).

*FTO* gene was identified in association with obesity and body mass regulation (20). It was in 2007 when studies by Gerken et al. revealed that the *FTO* encodes for a 2-Oxoglutarate-dependent Nucleic Acid Demethylase while the substrates were not known yet (21). In 2011, m6A was first shown to be the substrate of FTO in nuclear RNA and it was speculated that it plays a role in pre-RNA processing, transport or splicing (22). FTO was the first demethylase to be reported indicating m6A is a reversible modification. These findings brought a new dimension to the biological significance of m6A modifications. The other demethylase AlkBH5 was reported in 2013 and was shown to affect mRNA export and RNA metabolism (23).

**Figure 1:**
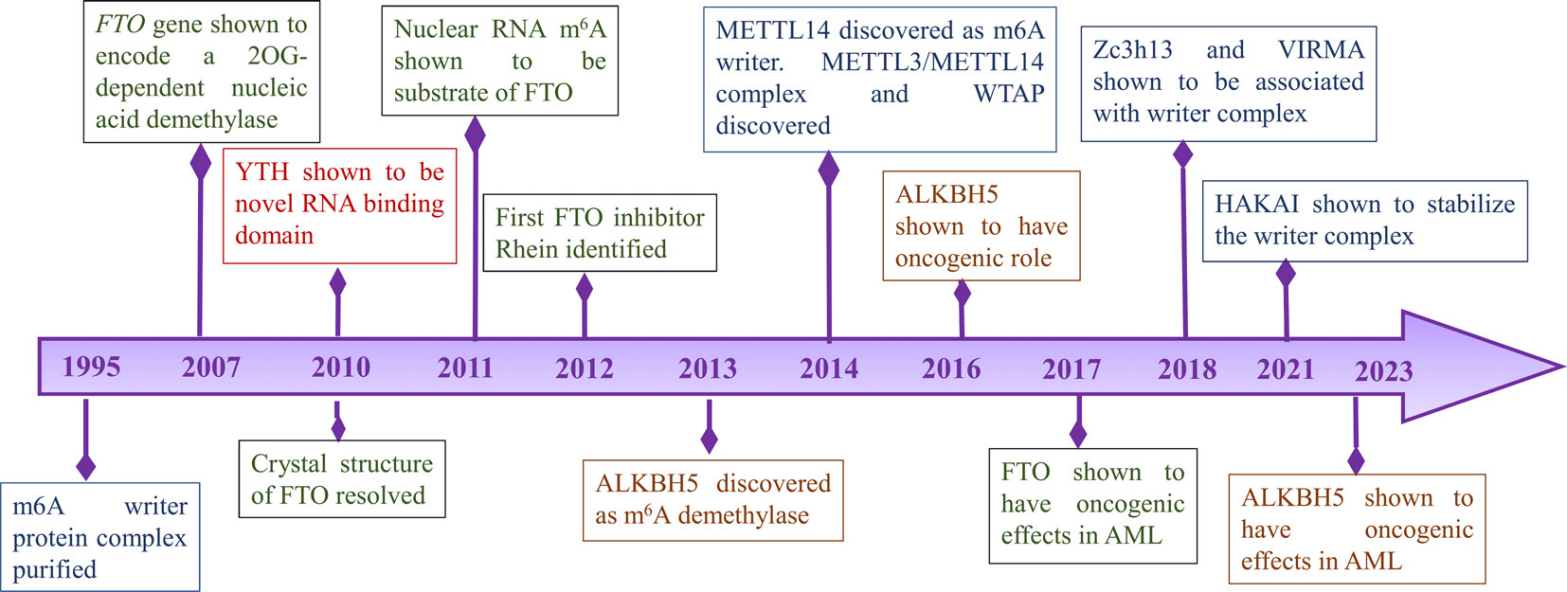
Timeline of important discoveries of RNA modification.

Discovery of demethylases highlighted the importance of m6A modification in various diseases. Further studies revealed ‘readers’ that are involved in various signaling pathways. Wang et al. discovered YTH domain family 2 (YTHDF2) as a reader protein that play a role in RNA metabolism (24). In 2016, another member of the YTH family protein YTHDF2 was identified as an alternative reader. YTHDF2 recruits the CCR4-NOT complex which is essential for the deadenylation of m6A-containing RNAs by CAF1 and CCR4 (25). Further, YTHDC1 and its role in mRNA splicing was identified in 2016 (26). In 2018, Insulin-like growth factor 2 (IGF2) mRNA binding proteins 1,2,3 (IGF2BP1/2/3) which promote stability of mRNA targets were identified as a new family of m6A readers (27). Important timelines of discoveries of different sub-units of RNA modifications are shown in **Figure 1**.

**Figure 2:**
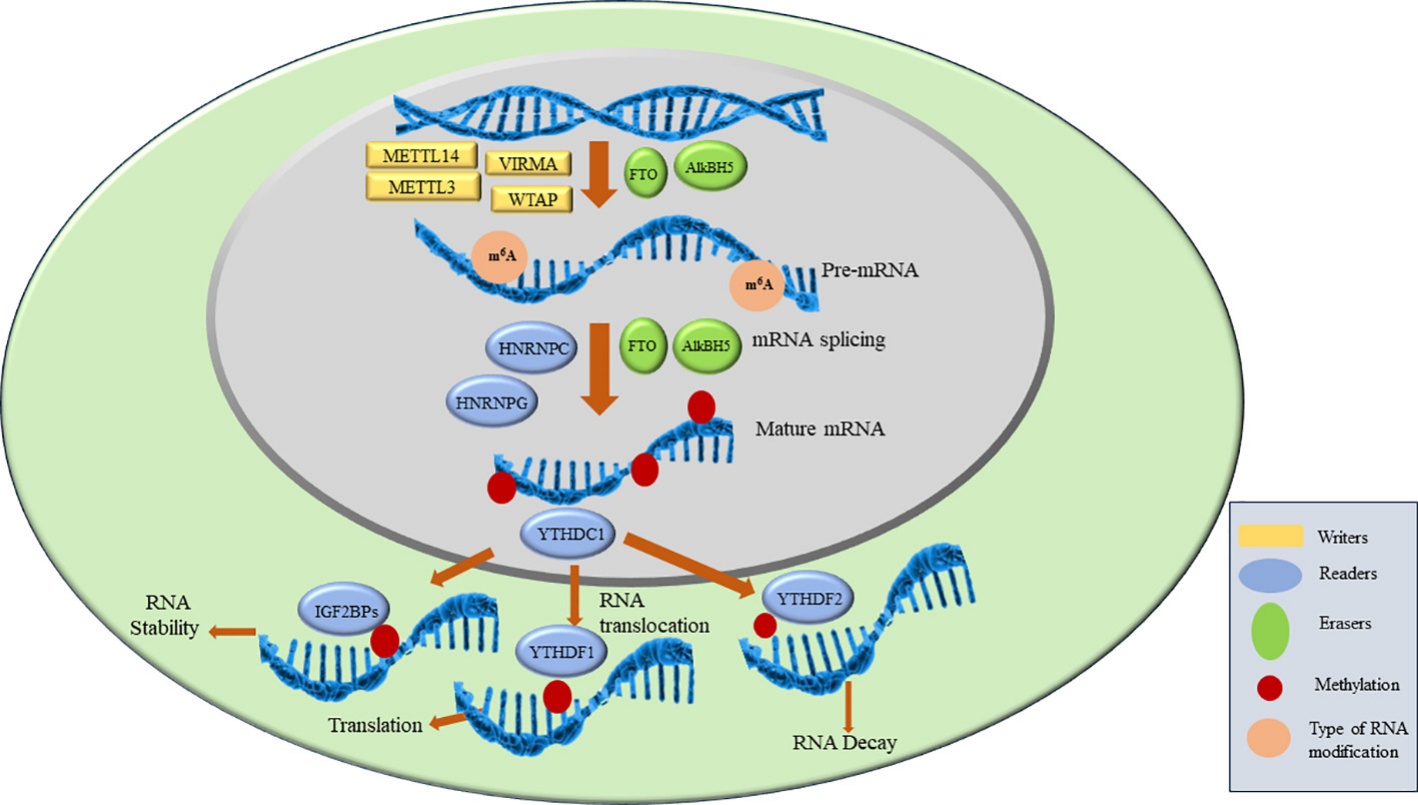
M6A RNA modification and proteins involved in the process.

As discussed above, the key players in m6A modifications are ‘writers’, ‘readers’ and ‘erasers’. Writers are methyltransferase complex proteins that help in the methylation of RNA and consist of METTL3, METTL14, WTAP, VIRMA, ZC3H13, RBM15/15B, HAKAI. Readers are proteins that recognize the m6A modification and regulate RNA stability and translation by binding to m6A. YTHDF1/2/3, YTHDC1/2, IGF2BP1/2/3, HNRNPC, HNRNPG and HNRNPA2B1 are reader proteins. Erasers are demethylase proteins that reverse the m6A modification. FTO and ALKBH5 are two demethylases reported so far (4, 5). The details of each of the above component of RNA modification is described in the next section and are shown in **Figure 2**.

### Writers

The writer complex consists of multiple subunits which add m6A to mRNA. This complex achieves methylation in a highly specific manner as only certain mRNAs are methylated and among those only certain consensus sites are methylated. However, the basis of this specificity remains poorly understood. After the discovery of the first methyltransferase METTL3 in 1994 more members are being added to date (11). The already reported and known members of Methyltransferase complex (MTC) includes METTL3, METTL14, VIRMA, WTAP, ZC3H13, RBM15/15B, HAKAI. METTL3 is the only catalytic subunit in MTC while others are associated proteins. METTL3 binds to METTL14 in 1:1 to form a dimer after which METTL3 becomes catalytically active and convert ‘A’ (adenosine) to m6A on mRNA. METTL14 is catalytically inactive as it lacks SAM binding site but it helps in activation of METTL3. This complex is localized in the nucleus but some reports show that stress can cause them to redistribute (28). Inactivation or deletion of METTL3 or METTL14 results in a 99% loss of total m6A modification in RNA indicating they play a potential role in achieving methylation (1). Associated proteins such as VIRMA, WTAP, ZC3H13, RBM15/15B, HAKAI have their specific functions. ZC3H13 helps in the nuclear localization of MTC and enhances m6A by bridging WTAP to the mRNA-binding factor Nito (29). Wilms tumor 1-associated protein (WTAP) is the adaptor protein of MTC which also lacks catalytic methylation. It interacts with METTL3 and METTL14 and helps in their recruitment of nuclear speckle. VIRMA is responsible for preferential m6A modification on mRNA at 3’UTR regions and stop codons. VIRMA mediates preferential m6A mRNA methylation in the 3’UTR and near the stop codon, while HAKAI affects m6A modification distributed in the 5’UTR and around the start codon (17). HAKAI also known as CBLL1, is a RING-finger type E3 ubiquitin ligase. It mediates the ubiquitination and endocytosis of the E-catherin complex which leads to cell-cell adhesion loss and increased cell mobility. It is also shown to regulate cell proliferation by affecting the binding of RNA targets to the PTB-associated splicing factors. Apart from these functions, HAKAI is the component of the methyltransferase complex in mammalian as well as plant cells. HAKAI is the component of MTC which is conserved in both drosophila and human and is a strong interactor of WTAP in mammalian cells (19). ZC3H13 enhances m6A processing through bridging WTAP to the mRNA-binding factor Nito while VIRMA directs m6A in 3′ UTR and near stop codon by recruiting the MTC to modulate region-selective methylation (30).

RBM 15 belongs to the split-end protein family. RBM15 and its paralog RBM15B were identified to interact with WTAP which showed that they might be a part of the methyltransferase complex (31). Later studies showed that they bind U-rich RNA consensus motifs and thus facilitate the recruitment of the MTC to specific sites in mRNA. RBM15 plays an important role in X chromosome inactivation by regulating m6A modification (32). Various studies show that RBM15 plays an important role in the recurrence of phyllodes tumour, laryngeal squamous cell carcinoma and hepatocellular carcinoma (33–35).

### Readers

Readers recognize the m6A and bind to them leading to different signaling pathways. They comprise of YTH domain-containing family protein 1/2/3 (YTHDF1/2/3), YTH domain-containing protein 1/2 (YTHDC1/2), insulin-like growth factor-binding protein 1/2/3 (IGF2BP1/2/3), heteronuclear ribonucleoproteins (including HNRNPC, HNRNPG and HNRNPA2B1 (6).

YTH domain family have a conserved m6A-binding domain. They bind at the RRm6ACH consensus sequence. YTHDF2 is the first characterized reader which is shown to accelerate the decay of m6A-modified transcripts by recruiting the CCR4-NOT deadenylase complex directly. YTHDF-1 interacts with the translation initiation factors which results in enhanced mRNA stability and protein synthesis. Further, interaction of YTHDF3 with YTHDF1 results in the enhanced RNA translation or RNA degradation. YTHDC1 also plays an important part in RNA splicing and export (26). Insulin-like growth factor 2 mRNA-binding proteins (IGF2BPs) including IGF2BP1–3 is recently identified as reader which bind to the m6A. They enhance the mRNA stability thus enhancing the RNA expressions. Another reader HNRNP A2/B1 (heterogeneous nuclear ribonucleoprotein A2/B1) binds to the RGAC motif on the RNA sites which results in m6A-dependent pre-miRNA processing events. It plays an important role in the mRNA stability by regulating pre-mRNA splicing in m6A dependent manner. It is shown to be upregulated in various cancers and is often associated with poor prognosis (36–38).

### Erasers

The m6A erasers are the demethylases that convert m6A to A. Though writers and readers were identified several decades ago, demethylases were discovered in 2011 when FTO was shown to have demethylase activity (22). ALKBH5 was discovered in 2013 and shows tissue-specific expression. Discovery of demethylases showed the first evidence of reversible post-transcriptional modification in mRNA (23). FTO was first defined as demethylase and belongs to the non-heme Fe (II)-2-oxoglutarate (2OG)-dependent dioxygenase AlkB family proteins that repair N-alkylated nucleobases by oxidative demethylation. It is present on chromosome 16q12.2 and contains 9 exons and 8 introns. It contains two domains namely the N-terminal domain and the C-terminal domain containing 32-326 residues and 327-498 residues, respectively. Both these domains interact with each other to destabilize the N-terminal domain to achieve demethylation. The unique structure of FTO helps in recognition of double-stranded DNA (dsDNA) and single-stranded DNA (ssDNA) or single-stranded RNA (ssRNA) (4). FTO shows binding to multiple types of RNAs, including mRNA, snRNA, and tRNA, and can demethylate m6A and N6, 2'-O-dimethyladenosine (m6Am) in mRNA, m6A in U6RNA, m6Am in snRNAs, and N1-methyladenosine (m1A) in tRNA. However, m6A is the most favourable nucleobase substrate of FTO (39).

FTO is differentially expressed in different age groups and also varies with body mass index. Its expression is high in foetal and adult tissues especially in brain. Single nucleotide polymorphisms in the FTO gene are shown to be associated with obesity and various cancers such as breast, prostate and lung cancer (40). Various studies indicate the role of FTO in the susceptibility to obesity as it increases adipogenesis by regulation of various adipogenic pathways and adipocyte differentiation (41). There are emerging studies that define the role of FTO in tumorigenesis and progression. Various molecular studies on the correlation between obesity and tumorigenesis yield contradictory results as most of the single nucleotide polymorphisms lie in the intronic regions of the *FTO* gene and intron 1 and 2 show the strongest association with obesity. These results can be explained by two hypotheses that either *FTO* introns act as auto-regulators or as cis-regulatory sites for adjacent genes. Knockout of the *FTO* gene in mice models shows reduced adipose tissues and lean body mass, suppressed mitochondria biogenesis and energy production by positively regulating mammalian target of rapamycin-peroxisome proliferator-activated receptor-g coactivator-1a (mTOR-PGC-1a) pathway. FTO is also linked to heart failure as its deficiency decreases cardiomyocytes contractile functions by accelerating Ca^2+^ decay and increasing sarcomere shortening during cardiac remodeling and repair (42). Thus, wide cellular role of FTO with major involvement in RNA demethylation process suggests its immense potential as candidate of research in various biological processes.

ALKBH5 is the second demethylase that was discovered in 2013 and is a member of the AlkB family. It also plays an important role in demethylation along with FTO and influences RNA stability, translation, splicing and export (23). ALKBH5 contains 395 amino acids. The catalytic core of ALKBH5 contains a DSBH domain consisting of 11 b strands (b1−b11) and 5 a helix (a1− a5). ALKBH5 binds to 2OG and metal ions in a conserved manner (4). Colocalization studies of ALKBH5 show that it is colocalized with mRNA-processing factors15 and thus regulates the translocation of mRNA from nuclear to cytoplasm. ALKBH5 can decrease the synthesis rate of nascent RNA and maintain global RNA stability by affecting RNA metabolism. ALKBH5 also shows differential expression in tissues and is located in nuclear speckles similar to FTO indicating different roles in the methylation. Knockout of ALKBH5 in cell lines showed increased export of target RNAs to the cytoplasm from the nucleus. Further, functional studies of ALKBH5 show that it mediates demethylation at 3’ UTR m6A. This demethylation results in cancer progression in various cancers such as breast cancers and glioblastoma. *FOXM1* which plays an important role in cell cycle and genomic stability is the downstream target of ALKBH5 in various cancers (43, 44). ALKBH5 also modulates the stability of 3’ UTR mRNAs in male germ cells (45). Recent studies have shown that ALKBH5 is expressed in the nucleus of adult mouse brain neurons, and is abundant in the cerebellum and olfactory bulb. The expression of ALKBH5 is usually high in the embryonic stages and decreases during the development of the brain indicating a probable role in brain development.

### Role of m6A RNA modification in leukemia

M6A modifications play an important role in progression, metastasis and chemoresistance in various cancers such as breast cancer, lung cancer, etc. The studies on leukemia are limited but they show that m6A plays a significant role in leukemia by influencing various cellular signaling and gene expression. A list of studies on expression of readers, writers and erasers in leukemia is listed in **Table 1**.

**Table 1:** Role of proteins involved in RNA modification in different leukemia and their target genes.

Protein	Cancer type	Expression	Target	Cohort details	References
Writers					
METTL3	Chronic myeloid leukemia	Overexpressed	Regulation of PES1 protein which acts as oncogene	K562, imatinib-resistant K562 imatinib-resistant, KCL22, LAMA84, NB4, HL-60, HEL and U937 cell lines	Ianniello et al., (46)
METTL3	Acute myeloid leukemia	Overexpressed	Association with chromatin and localization at transcriptional start site of active genes	MOLM13, THP-1, MV4-11, NOMO-1, HL-60, EOL-1, KG-1, RN2c, HEL, JURKAT, LOUCY, K562, NIH-3T3, SU-DHL-4, HT1080, SBC-3, DETROIT-562, FADU, SH-SY5Y and HT-29 cells. Rosa26Cas9/+, Flt3ITD/+; Rosa26Cas9/+ or moribund Npm1flox−cA/+; Flt3ITD/+ mice	Barbieri et al., (47)
METTL3/ METTL14	Acute myeloid leukemia	Overexpressed	Regulation of p53 signalling, cyclin dependent kinase inhibitor 1A (CDKN1A/p21), and mdm2.	89 patients, K562 and kasumi-1 cells	Sang et al., (48)
METTL3	Acute myeloid leukemia	Overexpressed	Promote AML homing and engraftment by regulating *ITGA4* mRNA and cause chemoresistance.	THP-1 and Kasumi-1 cells	Li et al., (49)
METTL3/ METTL14	B-cell acute lymphoblastic leukemia	Downregulated	Not defined	37 patients with ETV6/RUNX1(E/RUNX1) ‐positive acute lymphoblastic leukemia	Sun et al., (50)
WTAP	Acute myeloid leukemia	Overexpressed	Not defined	74 AML patients with non-M3 type and 19 normal controls	Yang et al., (51)
WTAP	Acute myeloid leukemia	Overexpressed	Regulating m6A level on lysine demethylase 4B (KDM4B)	33 t(8;21) AML patients, AML1::ETO-positive AML cell lines Kasumi-1 and SKNO-1	Shao et al., (52)
WTAP	Acute myeloid leukemia	Overexpressed	Regulating *c-Myc*	122 AML patients and eight normal subjects, K562 and MV4-11 cell lines	Naren et al., (53)
Readers					
YTHDF2	Acute myeloid leukemia	Overexpressed	Regulating AML1/ETO-HIF1α loop	33 patients with newly diagnosed t(8;21) AML, AML1/ETO-positive (Kasumi-1, SKNO-1-PGK, and U937-A/E) and AML1/ETO-negative (SKNO-1-siA/E and U937-MT) AML cell lines	Chen et al., (54)
YTHDF1	Acute myeloid leukemia	Overexpression	Regulating translation of *cyclin E2*	patient-derived xenograft models	Hong et al., (55)
IGF2BP1	Leukemia	Overexpression	Regulation of HOXB4, MYB, and ALDH1A1	K562, HL60, SKNO1, TANOUE, KASUMI1, REH, and MOLT16	Elcheva et al., (56)
IGF2BP1	B-Cell Acute Lymphoblastic Leukemia	Overexpression	Not defined	114 patients, 29 [1 altered cytogenetics, 13 other known translocations (11 BCR-ABL1, 1 E2A-PBX1, 1 MLL), 15 ETV6-RUNX1 positive], Reh cell lines	Sharma et al., (57)
IGF2BP2	Acute myeloid leukemia	Overexpressed	Regulation of MYC, GPT2, and SLC1A5	AML patients	Weng et al., (58)
Erasers					
FTO	Acute Myeloid Leukemia	Overexpressed	Inhibits all-trans-retinoic acid (ATRA)-induced AML cell differentiation, through regulating expression of targets such as ASB2 and RARA methylation.	109 human samples, K 562 and NB4 cells	Li et al., (59)
ALKBH5	Acute Myeloid Leukemia	Upregulated	Positively regulating the mRNA stability of *TACC3* transcripts leading to increased *TACC3* expression. *MYC* and *P21* levels also regulated indirectly.	U937, THP1, MV4–11, NOMO-1, MOLM13, NB4, MonoMac-6 (MMC6) cells. 136 TP53 wild-type and 14 mutant patients	Shen et al., (60)
ALKBH5	Acute Myeloid Leukemia	Overexpressed	ALKBH5 demethylate *ITPA* mRNA and increases its mRNA stability, leading to enhanced *ITPA* expression. Transcription factor TCF15 is responsible for the dysregulated expression of ALKBH5 in t (8;21).	Kasumi-1 cells, 12 t (8;21) AML samples	Li et al., (61)

### Writers

A study by Barbieri et al. using CRISPR screens showed METTL3 plays an essential role in the growth of leukemic cells in AML. METTL3 was shown to be associated with chromatin by localizing to the transcriptional start site of genes. *In vivo* studies show that downregulation of METTL3 results in suppression of leukemic cell progression and cell cycle arrest (47). Further in 2019, *METTL3* and *METTL14* expression was reported to be downregulated in *ETV6/RUNX1-*positive acute lymphoblastic leukemia as compared to the controls. *METTL3* and *METTL14* expression in relapse patients was also reported to be lower than the non-relapsed patients while the associated pathways were not studied (50). METTL3/METTL14 is also shown to be overexpressed in chronic myeloid leukemia and play an important role in the proliferation of leukemic cells. METTL3 plays an important role in ribosome biogenesis and translation. It also regulates the level of PES1 protein which is shown to have oncogenic role in breast cancer (46, 62). METTL3/METTL14 also regulates the expression of *p53*, *CDKN1a/p21* and *mdm2* thus having oncogenic effects in AML patients (48). METTL3 also plays a role in chemoresistance in AML by enhancing the half-life of *ITGA4* mRNA leading to increased expression of ITGA4 protein. This chemoresistance is shown to be reversed when cells are treated with METTL3 inhibitors (49).

Expression of WTAP is shown to be altered in AML patients and AML cell lines (63). WTAP expression is reported to be significantly higher in non-M3 Acute myeloid leukemia patients. WTAP is also shown to be highly expressed in *FLT3*-ITD mutated AML patients than in *FLT3*-ITD unmutated AML patients. *WTAP* mRNA expression is positively correlated with *WT1* expression (51). Further, WTAP has shown to be involved in proliferation, tumorigenesis, differentiation, cell cycle, and chemoresistance of AML cells. Moreover, WTAP has shown to regulate *c-Myc* expression which is oncogenic in nature (53). Overexpression of WTAP in *t(8;21)* AML patients is also linked to poor prognosis. Its activation by HIF1α results in reduced m6A levels on *KDM4B* transcripts which increase its degradation, thus enhancing the malignancy of leukemic cells (52). Further, studies in xenograft models show that growth rates of tumours in WTAP-knockdown cells are significantly reduced indicating WTAP plays an oncogenic role in AML.

### Readers

YTHDF2 is reported to be expressed across all AML samples but is significantly upregulated in *t(8;21)* acute myeloid leukemia patients as compared to non-*t(8;21)* controls. High expression of YTHDF2 is correlated to higher chances of relapse and high mortality in patients (54). It is shown to increase cell proliferation by regulating AML1/ETO-HIF1α loop. Differential expression of YTHDF2 in *t(8;21)* indicates to its potential to be used as a prognostic marker after more elaborative studies (54). Studies also show that YTHDF1 is also upregulated in AML and is required for the progression of leukemia. It is achieved by promoting the translation of cyclin E2. YTHDF2 is also being explored as a therapeutic target in AML (55). Apart from YTH family proteins, IGF2BP1 is shown to be highly expressed in various leukemic cell lines. Low levels of IGF2BP1 are correlated with better survival in AML patients as it results in reduced tumorgenicity in mice models. It regulates the expression of *ALDH1A1*, *HOXB4*, and *MYB* at the post-transcriptional level thus plays a role in leukemia stem cell phenotype (56). IGF2BP2 is also upregulated in AML and B-ALL. In AML, IGF2BP2 is shown to control the expression of *MYC*, *GPT2* and *SLC1A5*, although the specific signalling mechanisms in B-ALL are yet unidentified (57, 58).

### Erasers

FTO is shown to be abnormally regulated in *t(11q23)*/MLL-rearranged, *t(15;17)*/*PML-RARA*, *FLT3*-ITD, and/or *NPM1*-mutated AML phenotypes. It enhances cellular viability, and proliferation and inhibits apoptosis thus helping in leukemogenesis. FTO downregulation has been shown to result in an increase of all-trans-retinoic acid (ATRA)-induced AML cell differentiation. This is achieved by methylation regulation of two genes by FTO: *Ankyrin repeat and SOCS box protein 2 (ASB2) and retinoic acid receptor (RARA)*. This process also involves the interaction of FTO with YTHDF2 and YTHDF1; which indicates that the role of FTO is multifactorial (41, 64).

In studies involving the use of FTO inhibitors, it is reported that use of R-2-hydroxyglutarate (R-2HG) which was previously reported small-molecular inhibitor of FTO shows antileukemic effects in AML cell lines. R-2HG inhibits the FTO and thus increases the m6A levels on FTO oncogenic mRNA target *MYC/CEBPA*. These studies are also verified in the synergistic combination with first-line chemotherapy drugs such as daunorubicin or decitabine in murine models and further preclinical studies are required to check the therapeutic potentials (65).

ALKBH5 plays a critical role in autophagy, stem-cell renewal, cell proliferation, metastasis, and radioresistance in various cancers. Copy number alterations in the ALKBH5 are often reported in AML patients and are associated with inferior outcomes (66). MLST8, a common member of mTORC1 and mTORC2 protein complexes of the mTor pathway. mTor pathway is overexpressed in more than 70% of cancers. Studies show that targeting ALKBH5 by bioactive peptides results in decreased AML cell proliferation by repressing the demethylation of m6a in *MLST8/EIF4EBP1* mRNA axis. These findings suggest that RNA demethylase could be a potential therapeutic targets in leukemia (67).

## Distribution of m6A levels in cells of acute leukemia

The location of m6A sites on transcripts regulates m6A functions. Previous studies on transcriptome-wide m6A mapping of polyadenylated RNAs have shown that m6A modifications are enriched at 3′UTR region and near stop codon. Methylated RNA immunoprecipitation sequencing (MeRIP-seq) is one of the most commonly used tools for quantifying m6A modification at a specific site. Utilizing MeRIP-seq, studies have shown that that total m6A level are high in AML cells as compared to the normal hematopoietic progenitors. Deletion of METTL3 and METTL14 in embryonic cells results in reduced m6A levels affecting the differentiation of the cells (47, 68, 69). The reduction in m6A RNA by downregulating the expression of writers has shown to induce apoptosis in leukemic cells while having no effect on the normal hematopoietic progenitors. However, the detailed pathways studies associated with these observations are still awaited (70).

Methylation pattern of RNA is also shown to vary during the leukemia treatment. A study by Zhang et al. have shown that the number of m6A sites were decreased after azacytidine plus venetoclax treatment in patients bone marrow who achieved complete remission as compared to the AML bone marrows. Similar results were also obtained when HL-60 cell lines were treated with azacytidine. Further, downstream analysis has shown that most of the downregulated genes were protein coding indicating that they might play an important role in disease proliferation. In addition, *HPRT1*, *ANP32B*, and *SNRPC* were found to be linked to favourable outcomes in AML. These genes also showed a notable decrease in both m6A modification and expression (71).

## Role of m6A in various metabolic pathways in acute leukemia

Dysregulation of m6A modification can impact various metabolic pathways, contributing to the development and progression of acute leukemia. A study by Liu et al. has shown that m6A modifications are more prevalent on short-term hematopoietic stem and progenitor cells (ST-HSPC) than long-term hematopoietic stem (LT-HSPCs) and progenitor cells indicating its role in cell development. Further analysis of m6A methylome in AML cells has shown that the genes associated with fatty acid catabolic process-related genes shows higher m6A modification in leukemia initiating cells as compared to normal HSPCs. ATP-binding cassette subfamily D member 2 (ABCD2, also called ALDRP) which is involved in lipid metabolism was shown to be significantly high in AML patients as compared to healthy controls. Also, the increased expression of ABCD2 was shown to be correlated with poor prognosis. Further studies on AML cell lines MOLM13 and MV4-11 showed that ABCD2 plays a role in proliferation of AML cells (72). Similar study in AML has shown that IGFBP2 is upregulated in AML patients as well as in cell lines MV4-11, KASUMI, MOLM13, and THP1. Further *in-vivo* studies have shown that IGFBP2 is required for AML cell growth and PRMT6 is downstream target. The expression of *PMRT6* was also shown to be higher in AML patients and was correlated with poor overall survival in the patients. Targeting the expression of *PMRT* using inhibitor EPZ020411 resulted in decreased AML cell proliferation. MFSD2A which is sodium-dependent lysophosphatidylcholine symporter that is responsible for the uptake of docosahexaenoic acid (DHA) was found to be the downstream target of *PMRT6*. *PMRT6* was shown to inhibit the *MFSD2A* by catalyzing H3R2me2a modification (73). IGFBP2 is shown to play a role in regulation of glutamine metabolism by stabilizing the associated mRNA transcripts *MYC*, *GPT2*, or *SLC1A5* (58). ALKBH5 is also shown to modulate energy metabolism by m6A dependent manner. It controls OGDH RNA stability which is a key metabolic enzyme of TCA cycle (74).

## Role of m6A in various signalling pathways

Malignant cells are often characterized by the abrupt signalling pathways. M6A modification is associated with different signalling pathways in various cancers. PI3K/AKT pathway which is associated with cell growth, is shown to be regulated by m6A in many cancers. The alterations in *PI3K* or *AKT* genes or upstream signalling molecules result in uncontrolled cell growth and chemoresistance. Dysregulation of METTL3/14 is linked with PTEN expression which can activate PI3K/AKT pathway in pancreatic cancer and renal cell cancer (75, 76). The Janus kinase (JAK)-signal transducer and activator of transcription (JAK-STAT) is also associated with cell proliferation, differentiation and migration. SOCS protein negatively regulate the JAK-STAT signalling. SOCS2 is shown to be regulated by METTL3 and YTHDF2 in various cancers (77–79). This signalling is also shown to play an important role in T-cell homeostasis by regulating STAT signalling inhibitory proteins SOCS1, SOCS3 and CISH which further play a role in IL-2 signalling pathways in m^6^A dependent manner (64). p53 pathway plays an important role in cellular stress signalling. Aberrant expression of p53 is linked to various cancers. M6A modification regulate the genes such as *Mdm2*, *Mdm4* and *p21* which are involved in p53 signalling. Dysregulation of METTL3, METTL14, ALKBH5 and FTO is shown to affect the p53 signalling in various cancers leading to cancer progression (80–82).

## M6A as therapeutic target

**Figure 3:**
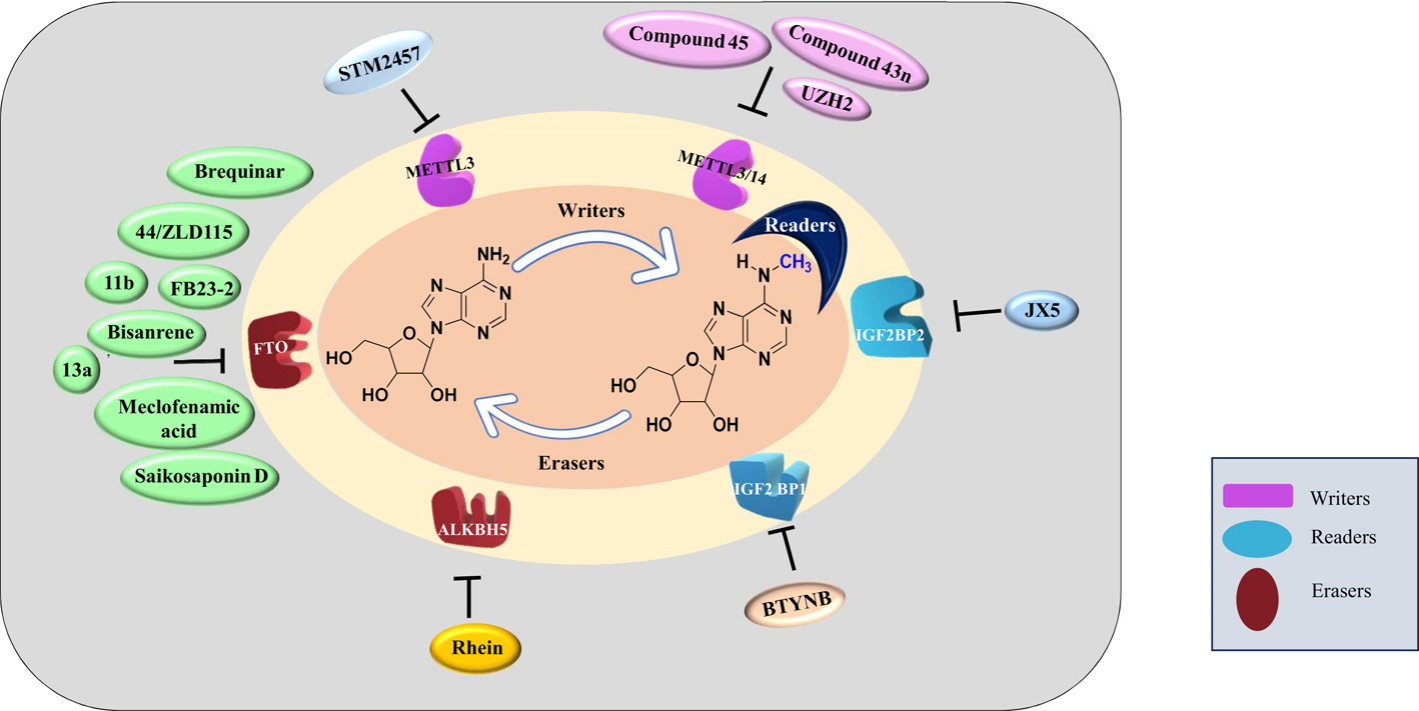
Inhibitors of m6A methylation complex targeting different components of RNA modification reported in different cancers.

The differential expression of ‘readers’, ‘writers’ and ‘erasers’ make them a suitable candidate for targeted therapy. The discovery of demethylases has revolutionized the research on RNA modifications. After this discovery, writers and readers also grabbed the attention of various researchers and various inhibitors targeting writers and readers were reported which are listed in **Table 2** and depicted in **Figure 3**. One of the first reports of inhibitors of ‘writers’ is that of STM2457 which shows selective inhibition of METTL3 and does not interfere with any other methyltransferases. These findings were confirmed by X-ray crystallography, surface plasmon resonance, and cellular thermal-shift assay. *In vitro* assays on MOLM-13 cells show decreased proliferation of cells and cell cycle arrest when treated with STM2457 while no such effects were seen on normal human umbilical cells and non-leukemic hematopoietic cell line HPC7. Similar results were seen on primary mice AML cells. Treatment with STM2457 resulted in decreased m6A levels on mRNA. Protein levels of SP1 and BRD4 which are substrates of METTL3 in AML were also reduced after STM2457 treatment. Further to decipher the detailed mode of action, MOLM-13 cells treated with STM2457 were subjected to m^6^A-specific methylated RNA immunoprecipitation (m^6^A-meRIP-seq) which revealed m6A associated DRACH motif as the top substrate. Further, *in vivo* studies using three human AML patient-derived xenograft showed that daily treatment with STM2457 resulted in decreased AML proliferation and prolonged survival in mice. Also, STM2457 did not show any toxicity on hematopoietic cells. Further trials are being done on STM2457 (9). β-elemene extracted from the Chinese medicinal plant *Curcuma Wenyujin* is also shown to inhibit METTL3 activity and have tumor suppressive effects. Various other studies show that it also targets *p21*, *p53*, *Myc*, *HTRA* etc. indicating that it has multiple targets of action (111, 112).

**Table 2:** Inhibitors of ‘readers’, ‘writers’ and ‘erasers’ reported in different cancers.

Inhibitor	Cancer type	Cohort	Target	References
Compound 45	AML	MOLM-13 AML cell line	METTL3/METTL14	Bedi et al., (83)
STM2457	AML, Non-small cell lung cancer	MOLM-13 AML cell line, non-leukemic haemopoietic cell line HPC7, xenograft model	METTL3	Xiao et al., (84); Yankova et al., (9)
Adenosine	–	Docking studies	METTL3	Bedi et al., (85)
UZH1a	–	MOLM-13, osteosarcoma U2OS cells and immortalized human embryonic kidney cell line HEK293T	METTL3	Moroz‐Omori et al., (86)
Elvitegravir	Esophageal squamous cell carcinoma (ESCC) cell lines	Currently used for treatment of HIV treatment. KYSE150, KYSE270, and EC9706 cells	METTL3	Liao et al., (87)
43n	Acute myeloid leukemia	MOLM-13, MOLM-14, THP-1, HL60, and K562	METTL3/METTL14	Lee et al., (88)
Compound 2p	–	HEK293T cells and AML MOLM-13 cells		
UZH2	–	MOLM-13 (acute myeloid leukemia) and PC-3 (prostate cancer) cell lines.	METTL3/METTL14	Dolbois et al., (89)
Quercetin	–	Human pancreatic carcinoma cell line MIA PaCa-2, Huh7 liver cancer cells	METTL3-METTL14	Du et al., (90)
Hakin-1	–	HT-29 and LoVo colorectal cancer cell lines, and *in vivo*, in a tumour xenograft mouse model,	HAKAI	Martinez-Iglesias et al., (91)
Ebselen	–	PC-3 cells, HEK293T cells	YTHDF proteins	Micaelli et al., (92)
BTYNB	Leukemia	Human HL60 and K562 cells	IGF2BP1	Jamal et al., (93)
JX5	T-cell acute lymphoblastic leukemia	Jurkat and Molt4 cells	IGF2BP2	Feng et al., (94)
Rhein	–	U87 cells	AlkB	Li et al., (95)
Meclofenamic acid	–	Human HeLa cells	FTO	Huang et al., (96)
18097	Breast cancer	Human cancer cells including MDA-MB-231, HeLa, HEK-293T, A549, A375 and mouse 3T3-L1 cells	FTO	Xie et al., (97)
44/ZLD115	Leukemia	MOLM13 cell lines	FTO	Xiao et al., (98)
FB23-2	AML	Leukemia cells NB4, U937, MV4-11, and ML-2, MONOMAC6, primary cells in xenotransplanted mice	FTO	Huang et al., (99)
FTO-043	gastric cancer cells	HEK293T cells, MRC-5 cells, U937 cells, NB4 cells, THP-1 cells and CCD 841 CoN cells	FTO	Huff et al., (100)
C6	esophageal cancer	KYSE-150, KYSE-270, TE-1, KYSE-510, and EC109 cell lines	FTO	Qin et al., (101)
13a	AML	MONOMAC6-transplanted NSG mice	FTO	Liu et al., (36)
20m	–	HepG2 cells	ALKBH5	Fang et al., (102)
Ena15 and Ena21	–	glioblastoma multiforme-derived cell lines	ALKBH5	Takahashi et al., (103)
11b	–	acute monocytic leukemia NOMO-1 cells	FTO	Prakash et al., (104)
Saikosaponin D	AML	NB4, Kasumi-1, K562, U937, HL60, SKNO-1, MV4-11, MOLM13, MOLM14, and mouse C1498 cells. C57BL/6N mice, ***in vivo*** studies in NU/NU Nude mice and immunodeficient NOD SCID mice	FTO	Sun et al., (105)
Diacerein	–	HeLa cells	FTO	Zhang et al., (106)
Bisantrene and Brequinar	AML	Human AML cell lines U937, THP1, NOMO-1, ML-2, NB4, MONOMAC 6 and MV4-11, AML patient-derived primary cells, Breast tumor cell lines ZR-75-1 and MDA-MB-231, pancreatic cancer cells Capan-1 and MIA PaCa-2, ‘human-in-mouse’ xeno-transplanation models and PDX models	FTO	Cully, (107); Su et al., (108)
Clausine E	–	–	FTO	Wang et al., (109)
Radicicol	–	–	FTO	Wang et al., (38)
MV1035	glioblastoma	U87-MG cells	AlkBH5	Malacrida et al., (110)

Rhein is the first natural molecule reported as an FTO inhibitor identified by using structure-based virtual screening. It competitively binds to the catalytic subunit of FTO (113). Meclofenamic acid is an anti-inflammatory drug that selectively inhibit FTO over ALKBH5. Due to poor pharmacodynamics and low efficacy, their analogues are being synthesized. FB23 and FB23-2 are recently developed analogues of Meclofenamic acid showing high efficacy against primary AML leukemic stem cells. However, their downstream targets are under study (99). Another study identified CS1 and CS2 as inhibitors of FTO by using in silico screening. They show significant inhibition of AML cells with IC50 much lower than FB23 and FB23-2 by activating apoptotic signaling and inhibiting the MYC pathway (108).

Computational modeling using the crystal structure of ALKBH5 identified 2-[(1-hydroxy-2-oxo-2- phenylethyl)sulfanyl]acetic acid and 4-{[(furan-2-yl)-methyl]amino}-1,2-diazinane-3,6-dione which showed inhibitory effects on leukemic cell lines by inhibiting ALKBH5 (8). A study published by Zhang et al. showed that bioactive peptide (BP) can downregulate ALKBH5 which results in decreased proliferation of leukemic cells in AML. Treatment with BP resulted in decreased ALKBH5 expression that increased apoptosis and inhibition of proliferation of leukemic cells *in vitro* and *in vivo* models. Further, *MLST8* and *EIF4EBP1* expression was also found to be downregulated and these two were identified as downstream targets of ALKBH5 by m6A sequencing. Downregulation of ALKBH5 results in reduced demethylation of these target mRNA resulting in inhibition of cell proliferation. Interestingly, they have also reported that combination therapy of cytarabine and BP demonstrated decreases tumor growth and increases survival rates in *in vivo* mice model (67).

According to the report published by Megan Cully, various companies are targeting RNA epigenetics. Compounds targeting METTL3 currently being developed by STORM and Gotham Therapeutics are likely to be under trails from 2021. STORM therapeutics was also aiming to put STM2457 in clinical trials in 2022 but the details are awaited (107).

Apart from using various compounds targeting the m6A regulators, CRISPR-Cas-based strategies are also being explored to develop molecular therapies. A study by Liu et al. has reported ‘m6A editing’ approach which uses the engineered CRISPR-Cas9 fused with m6A methyltransferase or erasers that can be programmed with a guide RNA to install or erase m6A modification (114). Further study by Wilson et al. used CRISPR-Cas13 and RNA methyltransferases to develop Targeted RNA methylation (TRM) system which can install m6A modification on endogenous RNA transcripts. TRM is shown to achieve install methylation in nucleus as well as cytoplasmic transcripts by using dCas13–M3nls and dCas13–M3M14nes respectively (115). Methylation pattern alteration of *HMBOX1* RNA in LnCAP cells using endonuclease-dead Cas9 protein (also known as dCas9) fused to the RNA demethylase ALKBH5 or METTL3 has shown that m6A modification plays an important role in determining the tumour suppressive function of *HMBOX1* (116).

While studies show the efficacy of various m6A modification inhibitors, detailed studies are required to study off-target effects, synergistic studies with various chemotherapy agents and enhanced effectiveness to utilize them in regimens.

## Future perspectives

The role of m6A modification has garnered significant attention as a promising therapeutic target for various cancers, including leukemia. Research has shown that the dysregulation of m6A-related proteins—such as m6A writers (e.g., METTL3, METTL14), erasers (e.g., FTO, ALKBH5), and readers (e.g., the YTHDF family)—is crucial in driving cancer cell proliferation, survival, chemoresistance, and relapse. In leukemia, these proteins influence the fate of leukemic stem cells, playing a key role in disease progression and therapy resistance.

One important consideration is the context-dependent expression and function of m6A-associated proteins across different cancers, and even within subtypes of the same malignancy. For instance, while METTL3 overexpression may promote oncogenesis in some leukemias, it could serve a different or even opposing function in other cancers. This variability highlights the need for a advanced approach that examines the tumor-specific expression profiles of m6A regulators, rather than adopting a uniform strategy for targeting m6A modifications across all cancers. A deeper understanding of the role of m6A machinery in different cancer subtypes is essential for the development of effective, personalized therapies.

Compounds targeting m6A regulators can be potential candidates for cancer therapy. These studies have shown high efficacy in *in vitro* and *in vivo* studies. M6A modification also influences how cancers respond to various treatments such as chemotherapy, radiotherapy, and immunotherapy. Utilizing m6A regulators in combination with these therapies could potentially enhance treatment outcomes and patient responses. Further, detailed pharmacological studies should be conducted to provide a deeper understanding of off-target effects, tissue distribution and stability.

Although targeting m6A modulators holds considerable potential, it also presents significant challenges. Since m6A modification is crucial in regulating normal physiological processes such as hematopoiesis, immune responses, and cellular differentiation, inhibiting these proteins could lead to unintended off-target effects and toxicity in healthy cells. This underlines the importance of developing selective targeting approaches that can distinguish between cancerous and healthy tissues, possibly by identifying specific vulnerabilities in cancer cells that are dependent on abnormal m6A regulation.

Moreover, the discovery of specific inhibitors or small molecules capable of modulating m6A-related proteins with precision is crucial. Any therapeutic strategy aimed at these proteins must be thoroughly evaluated to minimize interference with normal biological functions and reduce the risk of adverse side effects.

In summary, while targeting m6A-associated proteins represents a promising new direction in cancer treatment, including for leukemia, it is critical to further explore their cancer-specific roles. The development of safe, selective, and effective therapeutic strategies requires a comprehensive understanding of the differential expression and function of m6A regulators across various cancer types and subtypes, ensuring the therapeutic potential of m6A modulation is fully realized while minimizing associated risks.
